# Systemic Oxidative Stress Markers in Endometriosis: Elevated Advanced Glycation End Products and Sestrin 2 in Women with Ovarian Endometrioma

**DOI:** 10.3390/biomedicines14020405

**Published:** 2026-02-10

**Authors:** Nura Fitnat Topbas Selcuki, Kubra Bagci, Feyza Nur Tuncer, Cihan Kaya, Salih Yilmaz, Pinar Yalcin Bahat

**Affiliations:** 1 Department of Obstetrics and Gynecology, Istanbul Sisli Hamidiye Etfal Training and Research Hospital, University of Health Sciences Turkey, 34453 Istanbul, Türkiye; fitnat.topbas@gmail.com; 2 Obstetrics and Gynaecology, Private Clinic, 35220 Izmir, Türkiye; info@drkubrabagci.com; 3 Department of Genetics, Aziz Sancar Institute of Experimental Medicine, Istanbul University, 34093 Istanbul, Türkiye; 4 Department of Obstetrics and Gynaecology, Istanbul Aydin University, 34295 Istanbul, Türkiye; cihankaya@aydin.edu.tr; 5 Obstetrics and Gynaecology, Alle Clinic, 34746 Istanbul, Türkiye; drslhylmz@yahoo.com; 6 Department of Obstetrics and Gynaecology, Istanbul Yeni Yuzyil University, 34010 Istanbul, Türkiye; info@pinaryalcinbahat.com

**Keywords:** endometriosis, advanced glycation end products, sestrin 2, biomarker

## Abstract

**Background/Objectives:** Endometriosis is a chronic inflammatory disease characterized by ectopic endometrial tissue growth and is strongly associated with oxidative stress; however, systemic biomarkers reflecting this stress response remain limited. Advanced glycation end products (AGEs) promote oxidative and inflammatory signaling, while sestrin 2 (SESN2) is a stress-inducible protein involved in cellular redox homeostasis. This prospective case–control study aimed to evaluate serum AGEs and SESN2 levels in women with ovarian endometrioma and to assess their diagnostic performance. **Methods:** A total of 80 reproductive-aged women were enrolled, including 37 patients with ultrasonographically confirmed ovarian endometrioma and 43 healthy controls. Serum AGEs and SESN2 concentrations were measured using enzyme-linked immunosorbent assay. **Results:** Both biomarkers were significantly elevated in patients compared with controls (AGEs: 110.11 ± 33.35 vs. 91.70 ± 41.82 ng/mL, *p* = 0.007; SESN2: 9.32 ± 2.59 vs. 5.57 ± 1.52 ng/mL, *p* < 0.001). Receiver operating characteristic analysis demonstrated modest discriminatory ability for AGEs (AUC = 0.656), whereas SESN2 showed high diagnostic accuracy (AUC = 0.893), with 87.39% sensitivity and 86.05% specificity at an optimal cut-off value. Neither AGEs nor SESN2 levels were associated with lesion size, laterality, or pain symptoms. **Conclusions:** These findings provide the first evidence that circulating AGEs and SESN2 are elevated in ovarian endometrioma, supporting the role of systemic oxidative stress and stress-response pathways in endometriosis. SESN2, in particular, emerges as a promising biomarker candidate for disease presence, warranting further validation in larger and more diverse endometriosis cohorts.

## 1. Introduction

Endometriosis affects approximately 10% of women during their reproductive years and is characterized by endometrial-like tissue growing outside the uterine cavity [1]. The disease causes considerable morbidity through dysmenorrhea, dyspareunia, chronic pelvic pain (CPP), and infertility, yet its underlying mechanisms remain incompletely understood. One major challenge is the substantial diagnostic delay often 7–10 years from initial symptoms to diagnosis [2]. This diagnostic challenge is also reflected in recent clinical correspondence describing endometriosis being overlooked in unexpected presentations [3]. Over the years, diagnosis relied on laparoscopic detection of ectopic endometriotic loci with histopathological confirmation. However, in the latest European Society of Human Reproduction and Embryology (ESHRE) Endometriosis Guideline, an ultrasonographic detection of endometriosis has become the gold standard in diagnosis aiming to shorten this diagnostic delay by utilizing expert sonographists [4]. Multiple factors contribute to disease development, including hormonal imbalances, immune dysfunction, and genetic predisposition [5]. Among these, oxidative stress has gained increasing recognition in endometriosis pathogenesis [6]. Studies show that women with endometriosis have elevated markers of oxidative stress both systemically and in the peritoneal environment, with increased reactive oxygen species (ROS) production and reduced antioxidant defences [7,8]. This creates a pro-inflammatory milieu that is thought to promote ectopic tissue survival, proliferation, and neovascularization. Better understanding of the molecular mediators involved in these oxidative and inflammatory processes may shed light on disease mechanisms.

**Figure 1:**
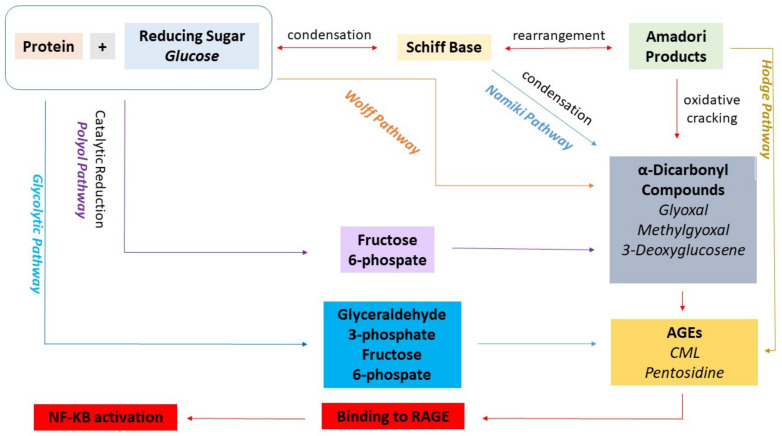
Formation of AGEs. Reducing sugars initially react with protein amino groups to generate reversible Schiff bases, which rearrange into stable Amadori products. These intermediates either undergo further modification via the Hodge pathway or are degraded into highly reactive dicarbonyl compounds that promote protein cross-linking and AGE formation. Reactive dicarbonyls are also produced through the Wolff, Namiki, and polyol pathways. Once formed, AGEs bind to their receptor RAGE, activating the NF-κB signaling pathway, enhancing reactive oxygen species (ROS) production, and triggering the release of pro-inflammatory mediators. (Diagram adapted from Chen et al.) [17].

Advanced glycation end products (AGEs) form when reducing sugars react non-enzymatically with proteins, lipids, or nucleic acids through the Maillard reaction [9,10]. These compounds accumulate both endogenously, particularly under hyperglycemic or oxidative stress conditions, and exogenously from dietary sources [11]. Once formed, AGEs bind to their receptor RAGE, triggering intracellular cascades that activate nuclear factor-κB, generate additional ROS, and release inflammatory mediators [12,13] (Figure 1). In reproductive medicine, AGEs have been studied primarily in the context of polycystic ovary syndrome, where elevated levels contribute to ovarian dysfunction, insulin resistance, and inflammation [14]. Links have also been demonstrated between AGEs and infertility, preeclampsia, and gestational diabetes [15,16]. However, few studies have examined AGEs in endometriosis, despite the well-documented role of oxidative stress in the disease.

**Figure 2:**
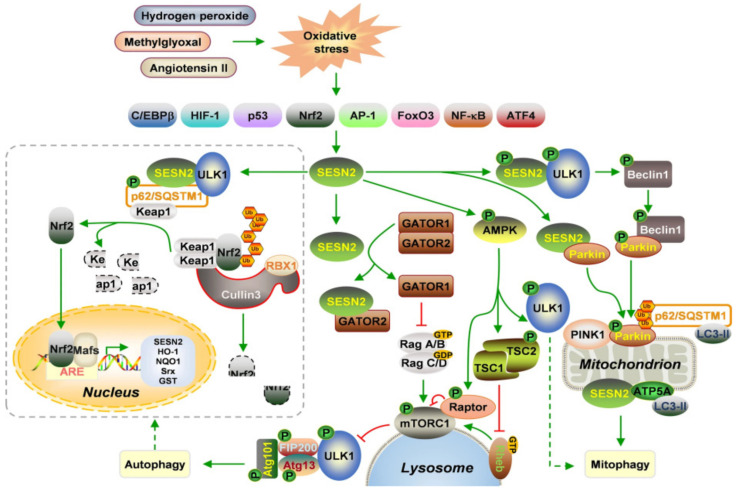
SESN2 action mechanism upon oxidative stress. The impact of oxidative stress relayed by the action of SESN2 through disparate pathways are depicted by Lu et al. In brief, SESN2 inhibits mTORC1 by binding GATOR2, releasing GATOR1 to inactivate Rag A/B and prevent mTORC1 lysosomal recruitment, thereby enabling ULK1 activation and autophagy initiation. SESN2 also suppresses mTORC1 indirectly through AMPK-dependent phosphorylation of Raptor and TSC2. In parallel, SESN2–ULK1 interaction promotes p62/SQSTM1-mediated Keap1 degradation, enhancing Nrf2 nuclear translocation and antioxidant gene expression. Additionally, SESN2 facilitates mitophagy by promoting ULK1–Beclin1–Parkin signaling, strengthening PINK1–Parkin interactions, or by recruiting LC3 to mitochondria via ATP5A [21].

Sestrins represent a family of stress-inducible proteins (SESN1, SESN2, and SESN3) that help cells respond to various stressors including DNA damage, oxidative injury, and metabolic disturbances [18,19]. They act through multiple pathways, regenerating peroxiredoxins, activating AMPK, inhibiting mTORC1, and modulating autophagy, to restore cellular balance, which is depicted by Lu et al. in Figure 2 [20,21]. SESN2, in particular, has attracted interest for its dual roles in different cancers: it suppresses tumor growth in colorectal and bladder malignancies, but can promote cell survival under certain stress conditions like nutrient deprivation [22,23,24]. Recently, attention has turned to SESN2 in benign gynecological conditions such as uterine leiomyomas and endometrial polyps as well pregnancy complications such as preeclampsia [25,26,27]. However, no studies to date have examined SESN2 in endometriosis.

Given the role of oxidative stress in endometriosis, we hypothesized that both AGEs and SESN2 might be dysregulated in this disease. AGEs could potentially amplify the inflammatory cascade through RAGE signaling, while SESN2 elevation might reflect a compensatory cellular stress response. Accordingly, this study was designed to evaluate serum AGE and SESN2 levels in women with ovarian endometrioma compared with healthy controls. In addition, we aimed to investigate their association with disease presence and clinical features, including lesion size, laterality, and symptom severity, in order to explore their potential as diagnostic biomarkers.

## 2. Material and Methods

### 2.1. Study Design and Participants

This prospective case–control study was conducted at the Department of Obstetrics and Gynecology, University of Health Sciences Turkey, Istanbul Kanuni Sultan Suleyman Training and Research Hospital. Women aged 18–50 years, who presented to the gynecology outpatient clinic and were diagnosed with ovarian endometrioma based on transvaginal ultrasonography (TVUS) were enrolled as the patient group. The control group consisted of age-matched women attending the same clinic during the same period, who had no ultrasonographic or clinical evidence of endometriosis or other pelvic pathology.

### 2.2. Inclusion and Exclusion Criteria

The inclusion criteria for the patient group were a confirmed diagnosis of ovarian endometrioma on TVUS and being of reproductive age. Controls were required to have normal gynecological examination and ultrasonography findings.

Exclusion criteria for both groups included ongoing pregnancy; history of malignancy; diabetes mellitus; chronic inflammatory, hepatic, renal, or neurological disease; polycystic ovary syndrome; smoking; blood transfusion within the previous six months; hormone replacement therapy or hormonal treatment within the past year; and use of medications known to affect oxidative stress or metabolic pathways, including statins, angiotensin-converting enzyme inhibitors, angiotensin receptor blockers, calcium channel blockers, and thiazolidinediones. Additionally, women experiencing dysmenorrhea, dyspareunia and CPP of any severity were excluded from the control group to minimize the likelihood of undiagnosed superficial endometriosis, which may not always be detected on TVUS examination.

### 2.3. Clinical Assessment

Demographic and reproductive characteristics, including age, body mass index (BMI), gravidity, parity, abortus, and curettage history, were recorded for all participants. Pain-related symptoms were assessed using a visual analogue scale (VAS) for dysmenorrhea. The presence of CPP and dyspareunia was documented based on patient self-report. CPP was defined as non-cyclic pelvic pain persisting for ≥6 months, independent of pain severity, while dyspareunia was defined as the presence of any pain during sexual intercourse, regardless of severity or frequency, based on self-report during clinical evaluation. TVUS was performed using a high-frequency transvaginal probe by an experienced gynecologist blinded to patients’ medical history and existing laboratory tests. Ovarian endometriomas were measured in three perpendicular planes, and cyst volume was calculated using the ellipsoid formula. Laterality (unilateral or bilateral involvement) was recorded.

### 2.4. Blood Sample Collection and Laboratory Analysis

Venous blood samples were collected from all participants on the day of examination. Samples were centrifuged within one hour of collection at 1000× *g* for 15 min at 2–8 °C. Serum aliquots were immediately separated and stored at −80 °C until analysis.

Serum advanced glycation end products (AGEs) and sestrin 2 (SESN2) concentrations were measured using commercially available sandwich enzyme-linked immunosorbent assay (ELISA) kits, according to the manufacturers’ instructions (Mybiosource, San Diego, CA, USA; Cat. No: MBS760249 and MBS267540). All samples were analyzed in duplicates, and the same assay batches were used for patient and control samples to minimize inter-assay variability. The intra- and inter-assay coefficients of variation were within acceptable limits, as specified by the manufacturers.

### 2.5. Statistical Analysis

Statistical analyses were performed using NCSS (Number Cruncher Statistical System) 2007 Statistical Software (East Kaysville, Utah, USA). Descriptive statistics, including mean, standard deviation, median, and interquartile range (IQR), were used to summarize the data. The distribution of variables was assessed using the Shapiro–Wilk normality test. Comparisons between two independent groups were conducted using the independent *t*-test for normally distributed variables and the Mann–Whitney U test for non-normally distributed variables. Categorical variables were compared using the chi-squared test. To evaluate the discriminatory performance of serum AGEs (ng/mL) and SESN2 (ng/mL) for the presence of endometrioma, receiver operating characteristic (ROC) curve analyses were performed. Areas under the curve (AUCs) were calculated, and optimal cut-off values were determined based on sensitivity, specificity, positive predictive value, negative predictive value, and positive likelihood ratio (LR+). No internal resampling (e.g., cross-validation or bootstrapping) or external validation cohort was used; therefore, ROC-derived cut-off values and performance estimates should be interpreted as exploratory. A two-sided *p*-value < 0.05 was considered statistically significant.

## 3. Results

The study included 80 women, comprising 43 controls and 37 patients with endometrioma. Baseline demographic and reproductive characteristics are summarized in Table 1. Mean age was comparable between the control and patient groups (39.14 ± 6.64 vs. 34.51 ± 6.42 years, *p* = 0.09). Similarly, BMI did not differ significantly between groups (21.92 ± 1.85 vs. 21.72 ± 2.03 kg/m^2^, *p* = 0.652). Gravidity, abortus, and curettage history were also comparable. Parity was modestly, but significantly higher in the patient group compared with the controls (1.89 ± 0.61 vs. 1.42 ± 0.88, *p* = 0.015).

Pain characteristics, endometrioma features, and serum AGEs and SESN2 levels are summarized in Table 2. Among patients, dysmenorrhea severity was high, with a mean VAS score of 5.73 ± 1.33 and a median score of 6 (IQR 5–6.5). CPP was reported by 29.7% of patients, while dyspareunia was present in 13.5%. Adenomyosis was identified in a single patient (2.7%). Bilateral ovarian endometrioma was observed in 16.2% of cases, whereas the majority had unilateral involvement. Mean ovarian endometrioma volume was 5.98 ± 2.16 cm^3^ for right-sided lesions and 4.05 ± 1.24 cm^3^ for left-sided lesions. In parallel, serum AGEs and SESN2 concentrations were significantly higher in the patient group compared with controls, with mean AGE levels of 110.11 ± 33.35 ng/mL versus 91.70 ± 41.82 ng/mL (*p* = 0.007) and mean SESN2 levels of 9.32 ± 2.59 ng/mL versus 5.57 ± 1.52 ng/mL (*p* < 0.001), respectively. A statistically significant positive correlation was observed between AGEs and SESN2 levels (r = 0.286, *p* = 0.01) (Table 3).

**Figure 3:**
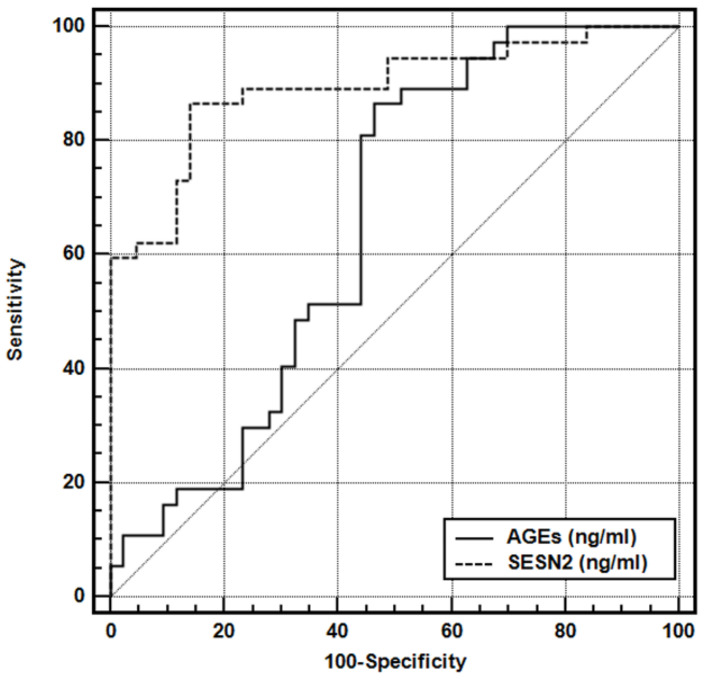
Receiver operating characteristic (ROC) curves for AGEs and SESN2.

The diagnostic performance of serum AGEs and SESN2 levels for the presence of endometrioma was evaluated using ROC curve analysis (Table 4, Figure 3). The AUC for AGEs was 0.656 (95% CI 0.542–0.759, *p* = 0.011), indicating modest discriminatory ability. At an optimal cut-off value of >77.66 ng/mL, AGEs demonstrated a sensitivity of 86.49% and a specificity of 53.49%, with a positive predictive value of 61.58%, a negative predictive value of 82.19%, and a positive likelihood ratio (LR+) of 1.86, corresponding to a 1.86-fold higher likelihood of endometrioma in patients with AGE levels above this threshold. In contrast, SESN2 showed a significantly higher diagnostic accuracy, with an AUC of 0.893 (95% CI 0.804–0.951, *p* < 0.001). Using a cut-off value of >6.88 ng/mL, SESN2 achieved a sensitivity of 87.39% and a specificity of 86.05%, with a positive predictive value of 84.24%, a negative predictive value of 88.28%, and an LR+ of 6.20, indicating a substantially increased likelihood of endometrioma among patients with elevated SESN2 levels.

Subgroup analyses within the patient cohort are summarized in Table 5. When patients were stratified according to ovarian endometrioma volume (<6 cm^3^ vs. ≥6 cm^3^), neither AGEs nor SESN2 levels differed significantly between the groups (AGEs: *p* = 0.413; SESN2: *p* = 0.854). Similarly, serum AGEs and SESN2 concentrations did not differ significantly between patients with unilateral versus bilateral endometrioma (AGEs: *p* = 0.359; SESN2: *p* = 0.903). When patients were categorized based on the presence of pain symptoms (dysmenorrhea, dyspareunia and/or chronic pelvic pain), mean AGEs and SESN2 levels were numerically higher in the pain-positive group; however, these differences did not reach statistical significance (AGEs: *p* = 0.211; SESN2: *p* = 0.129).

## 4. Discussion

This study provides the first evidence that both serum AGEs and SESN2 are significantly elevated in women with ovarian endometrioma compared to healthy controls. Our findings demonstrate that AGE levels were modest, but significantly higher in patients (110.11 ± 33.35 ng/mL) than controls (91.70 ± 41.82 ng/mL, *p* = 0.007), while SESN2 showed more pronounced elevation (9.32 ± 2.59 versus 5.57 ± 1.52 ng/mL, *p* < 0.001). SESN2 exhibited a good diagnostic performance with an AUC of 0.893, whereas AGEs showed more modest discriminatory ability (AUC 0.656). Notably, neither biomarker correlated with endometrioma volume, laterality, or pain symptoms in subgroup analyses, suggesting these markers reflect disease presence rather than specific phenotypic characteristics.

While the involvement of AGEs in endometriosis pathogenesis has been suggested, only limited research has directly examined this relationship. Fujii et al. measured soluble RAGE (sRAGE), vascular endothelial growth factor (VEGF), and carboxymethyl lysine (CML), a specific AGE, in plasma, follicular fluid, and peritoneal fluid of women with and without endometriosis [28]. Their findings revealed that while plasma levels were similar between the groups, follicular fluid from endometriosis patients showed significantly elevated sRAGE and VEGF, suggesting local AGE-RAGE axis activation in the ovarian microenvironment. More recently, Smyk et al. demonstrated that women with endometriosis exhibit increased skin AGE accumulation alongside endothelial dysfunction and arterial stiffness, findings that may explain the elevated cardiovascular disease risk observed in this population [29,30,31]. However, systematic examination of circulating serum AGE levels in endometriosis has remained largely unexplored. Our study addresses this gap by demonstrating for the first time that serum AGE levels are significantly elevated in women with endometriosis, extending previous observations from local compartments to the systemic circulation.

AGEs form through non-enzymatic glycation reactions that are accelerated under conditions of oxidative stress and inflammation, both known features of endometriosis [9,10]. Several mechanisms may explain increased AGEs in women with endometriosis. First, the disease is characterized by elevated peritoneal fluid iron concentrations due to retrograde menstruation and hemolysis of red blood cells [7,8]. Free iron catalyzes Fenton reactions, generating hydroxyl radicals and superoxides that promote oxidative modifications of proteins and lipids, thereby accelerating AGE formation. Second, the chronic inflammatory environment in endometriosis, marked by elevated pro-inflammatory cytokines such as IL-1β, IL-6, and TNF-α, creates sustained oxidative stress that could favor AGE accumulation [6,8]. Third, activated macrophages in the peritoneal cavity generate reactive oxygen species during phagocytosis of cellular debris and apoptotic endometrial cells, further contributing to oxidative damage [7,9]. The modest diagnostic performance of AGEs (AUC 0.656) suggests that although AGEs are elevated in endometriosis, they may lack sufficient specificity, as AGE accumulation occurs in multiple conditions associated with oxidative stress and inflammation, including diabetes, cardiovascular disease, and aging [10,11,12].

Several mechanisms could explain SESN2 elevation in endometriosis. First, SESN2 responds to oxidative stress by regenerating overoxidized peroxiredoxins, thereby maintaining antioxidant defenses [20]. The chronic oxidative environment in endometriosis, characterized by elevated ROS in peritoneal fluid and reduced antioxidant capacity, would be expected to trigger SESN2 upregulation as a compensatory response [7,8]. Second, SESN2 activates the AMPK pathway while suppressing mTORC1, thereby modulating cellular metabolism in response to energy stress [18,20]. Endometriotic lesions experience metabolic stress due to their ectopic location, fluctuating hormonal environment, and inflammatory milieu, which could drive SESN2 induction. Third, SESN2 is induced in a p53-dependent manner following DNA damage [20]. The oxidative stress in endometriosis can cause DNA damage in both eutopic and ectopic endometrium, potentially triggering SESN2 expression [6,8].

The superior diagnostic performance of SESN2 compared to AGEs in our study deserves consideration. SESN2 demonstrated an AUC of 0.893 with balanced sensitivity (87.39%) and specificity (86.05%), whereas AGEs showed lower specificity (53.49%) despite high sensitivity (86.49%). This difference may reflect the distinct biological roles of these molecules. AGEs accumulate in multiple conditions involving hyperglycemia, aging, and oxidative stress, limiting their specificity for endometriosis [10,11]. In contrast, SESN2 elevation may more specifically indicate the type of cellular stress present in endometriosis and gynaecological conditions involving the uterus [25,26]. The positive likelihood ratio of 6.20 for SESN2 indicates that elevated levels substantially increase the probability of endometriosis, suggesting potential clinical utility pending validation in larger studies.

Importantly, neither AGEs nor SESN2 levels correlated with endometrioma volume, laterality, or pain symptoms in our subgroup analyses. The absence of a significant correlation suggests that these biomarkers are more indicative of disease presence than of disease burden or severity of symptoms. Similar findings have been reported in the leiomyoma study, where SESN2 levels did not correlate with myoma volume or patient demographics [25]. Several interpretations are possible. First, once systemic stress response pathways are activated, circulating biomarker levels may plateau and not proportionally increase with lesion size. Second, the oxidative stress and inflammatory response in endometriosis may be more dependent on disease biology and host factors than on the physical extent of the condition [6,7]. The absence of an association with pain symptoms should be interpreted cautiously, as it may reflect limited clinical phenotyping rather than true biological independence. Pain in endometriosis is multifactorial and may depend on lesion depth, nerve involvement, central sensitization, and pain chronicity, factors that were not comprehensively characterized in this study [1]. More granular phenotyping of pain domains and disease subtypes may be required to clarify potential relationships between circulating stress markers and symptom burden.

From a pathophysiological perspective, our findings support the concept that endometriosis induces systemic alterations in oxidative stress and cellular stress response pathways [6,8,9]. The elevation of both AGEs and SESN2 suggests an imbalance between oxidative damage and compensatory protective mechanisms. AGEs represent accumulated oxidative damage to biomolecules [9,10], while SESN2 reflects the cellular attempt to counteract such damage [18,20]. This dual elevation indicates that despite upregulation of protective stress response proteins like SESN2, oxidative damage still results in the form of elevated serum AGE levels. Whether SESN2 elevation represents an adequate or insufficient compensatory response remains to be determined. Studies in cancer biology have shown that while SESN2 can have tumor-suppressive effects, it can also promote cell survival under stress, potentially contributing to disease persistence [22,23]. In endometriosis, SESN2 might similarly have dual roles; protecting cells from oxidative damage while potentially facilitating survival of ectopic endometrial tissue.

Several limitations of our study should be acknowledged. First, while the sample size was sufficient for detecting the observed differences, it may have limited power to assess correlations in subgroup analyses. Larger-scale studies are needed to clarify whether biomarker levels are associated with specific disease phenotypes. Second, we measured total serum AGEs rather than specific AGE subtypes, which may have different biological activities and disease associations. In addition, ELISA-based AGE measurements are not fully standardized across manufacturers and may capture heterogeneous AGE epitopes, limiting comparability across studies. More broadly, “total AGEs” represents a chemically diverse class rather than a single molecular species, which reduces molecular specificity and complicates mechanistic interpretation. Future studies should consider quantifying defined AGE adducts using more specific analytical approaches to improve biological interpretability and reproducibility. Third, we included only women with ovarian endometrioma, the most easily diagnosed form of endometriosis on ultrasound. Whether AGEs and SESN2 are similarly elevated in other forms of endometriosis (superficial peritoneal or deep infiltrating disease) requires investigation. For such a study design a laparoscopic visualisation and staging of disease would be required to clearly define disease stage and detect superficial endometriosis. Fourth, this was a cross-sectional study that cannot establish causality or temporal relationships. Longitudinal studies would be valuable to determine whether biomarker levels change with disease progression or treatment. Fifth, we excluded women with conditions known to affect AGE or SESN2 levels (diabetes, smoking, certain medications), which limits generalizability, but was necessary to isolate the effect of endometriosis. Real-world validation in more heterogeneous populations would be important. Seventh, while we attempted to exclude women with superficial endometriosis from controls by excluding those with pain symptoms, some minimal disease may have gone undetected, potentially leading to conservative estimates of group differences. Finally, we did not assess other oxidative stress markers or inflammatory cytokines that might provide additional context for interpreting AGEs and SESN2 elevations.

Despite these limitations, this study makes several important contributions. We provided the first evidence that both AGEs and SESN2 are elevated in endometriosis, extending observations from other gynecological conditions to this common disease. The elevation of these markers supports the importance of oxidative stress in endometriosis pathophysiology. The good diagnostic performance of SESN2 is particularly noteworthy, though whether this translates to clinical utility requires validation in independent cohorts and comparison with currently used diagnostic approaches. Most importantly, these findings highlight that endometriosis induces systemic metabolic and oxidative alterations that can be detected in peripheral blood, opening avenues for both mechanistic research and potential biomarker development.

## 5. Conclusions

This study offers proof-of-concept evidence that circulating AGEs and SESN2 are elevated in women with ovarian endometrioma, supporting systemic oxidative-stress-related alterations in this endometriosis phenotype. Although SESN2 showed strong discrimination in this selected ultrasound-defined OMA cohort, it cannot yet be considered a diagnostic biomarker, especially for non-OMA phenotypes or for routine clinical use in more heterogeneous, real-world populations. Larger, externally validated studies spanning the full spectrum of endometriosis are needed to determine clinical relevance.

## Figures and Tables

**Table 1:** Demographic and reproductive characteristics of the study population.

		Control Group (n = 43)	Patient Group (n = 37)	*p*-Value
Age (years)	Mean ± SD	36.81 ± 5.58	34.51 ± 6.42	0.09
BMI	Mean ± SD	21.92 ± 1.85	21.72 ± 2.03	0.652
Gravidity	Mean ± SD	2.44 ± 1.65	2.95 ± 0.91	0.143
	Median (IQR)	3 (1–3)	3 (2–3.5)	
Parity	Mean ± SD	1.42 ± 0.88	1.89 ± 0.61	0.015
	Median (IQR)	2 (1–2)	2 (1.5–2)	
Abortus	Mean ± SD	0.72 ± 1.01	0.59 ± 0.83	0.804
	Median (IQR)	0 (0–1)	0 (0–1)	
Curettage	Mean ± SD	0.26 ± 0.54	0.46 ± 0.65	0.102
	Median (IQR)	0 (0–0)	0 (0–1)	

BMI: body mass index, IQR: interquartile range.

**Table 2:** Pain characteristics, endometrioma features, and serum AGEs and SESN2 levels.

		Control Group (n = 43)		Patient Group (n = 37)		*p*-Value
Dysmenorrhea VAS Score	Mean ± SD	0 ± 0		5.73 ± 1.33		-
	Median (IQR)	0 (0–0)		6 (5–6.5)		
CPP	None	43	100.00%	26	70.27%	-
	Present	0	0.00%	11	29.73%	
Dyspareunia	None	43	100.00%	32	86.49%	-
	Present	0	0.00%	5	13.51%	
Adenomyosis	Absent	43	100.00%	36	97.30%	-
	Present	0	0.00%	1	2.70%	
Bilateral OMA	None	-	-	31	83.78%	-
	Present	-	-	6	16.22%	
OMA Volume (cm^3^)	Mean ± SD	Right OMA		5.98 ± 2.16		-
	Mean ± SD	Left OMA		4.05 ± 1.24		-
AGEs (ng/mL)	Mean ± SD	91.7 ± 41.82		110.11 ± 33.35		0.007
SESN2 (ng/mL)	Mean ± SD	5.57 ± 1.52		9.32 ± 2.59		<0.001

IQR: interquartile range; CPP: chronic pelvic pain; OMA: ovarian endometrioma.

**Table 3:** Evaluation of correlation between AGEs and SESN2.

		AGEs (ng/mL)
SESN2 (ng/mL)	r	0.286
	*p*	0.01
	N	80

**Table 4:** Diagnostic performance of AGEs and SESN2 for endometrioma.

Marker	AUC (95% CI)	*p*-Value	Cut-Off	Sensitivity (%)	Specificity (%)	PPV	NPV	LR (+)
AGEs (ng/mL)	0.656 (0.542–0.759)	0.011	>77.66	86.49	53.49	61.58	82.19	1.86
SESN2 (ng/mL)	0.893 (0.804–0.951)	<0.001	>6.88	87.39	86.05	84.24	88.28	6.20

CI: confidence interval; PPV: positive predictive value; NPV: negative predictive value; LR: likelihood ratio.

**Table 5:** Sub-phenotype analysis of serum AGEs and SESN2 levels in patients with endometriosis.

	OMA Volume < 6	OMA Volume > 6	*p*-Value
AGEs (ng/mL)	108.41 ± 31.94	117.46 ± 34.46	0.413
SESN2 (ng/mL)	9.24 ± 3.05	9.4 ± 2.09	0.854
	Bilateral OMA Absent	Bilateral OMA Present	*p*-value
AGEs (ng/mL)	110.59 ± 34.18	124.31 ± 25.74	0.359
SESN2 (ng/mL)	9.29 ± 2.8	9.44 ± 1.09	0.903
	Pain Absent	Pain Present	*p*-value
AGEs (ng/mL)	108.35 ± 34.32	123.37 ± 28.51	0.211
SESN2 (ng/mL)	8.89 ± 2.51	10.32 ± 2.61	0.129

## Data Availability

The data presented in this study are available on request from the corresponding author. The data are not publicly available because of Turkish Personal Data Protection Law no. 6698.
